# Impact of direct ICU admission of pneumococcal meningitis in France: a retrospective analysis of a French medico-administrative (PMSI) database

**DOI:** 10.1186/s13613-023-01239-1

**Published:** 2024-01-27

**Authors:** Michael Thy, Claire Dupuis, Arthur Mageau, Bruno Mourvillier, Lila Bouadma, Stéphane Ruckly, Anne Perozziello, Andrey Strukov, Damien Van-Gysel, Etienne de Montmollin, Romain Sonneville, Jean-François Timsit

**Affiliations:** 1 Medical and Infectious Diseases ICU, Bichat Claude Bernard University Hospital, Université Paris Cité, AP-HP Paris, France; 2 EA7323, Pharmacology and Drug Evaluation in Children and Pregnant Women, Université Paris Cité, Paris, France; 3 Department of Intensive Care Medicine, Gabriel-Montpied University Hospital, Clermont-Ferrand, France; 4 UMR 1137-IAME Team 5–DeSCID: Decision SCiences in Infectious Diseases control and care INSERM Université Paris Cité, 75018 Paris, France; 5 Medical Intensive Care Unit, Robert Debré University Hospital, Reims, France; 6 Department of Medical Information, Bichat Claude Bernard University Hospital, AP-HP, Université Paris Cité, Paris, France

**Keywords:** Pneumococcal meningitis, ICU, Sepsis, Direct admission, Mortality

## Abstract

**Background:**

Current guidelines for adult patients with pneumococcal meningitis (PM) recommend initial management in intermediate or intensive care units (ICU), but evidence to support these recommendations is limited. We aimed to describe ICU admission practices of patients with PM.

**Methods:**

We conducted a retrospective analysis of the French medico administrative database of consecutive adult patients with PM and sepsis criteria hospitalized between 2011 and 2020. We defined two groups, “Direct ICU” corresponding to a direct ICU admission and “Delayed ICU” corresponding to a secondary ICU admission.

**Results:**

We identified 4052 patients hospitalized for a first episode of PM, including 2006 “Direct ICU” patients (50%) and 2046 “delayed ICU” patients (50%). The patients were mainly males [*n* = 2260 (56%)] with median age of 61 years [IQR 50–71] and a median Charlson index of 1 [0–3]. Among them, median SAPS II on admission was 46 [33–62], 2173 (54%) had a neurological failure on admission with 2133 (53%) in coma, 654 (16%) with brainstem failure, 488 (12%) with seizures and 779 (19%) with focal signs without coma. PM was frequently associated with pneumonia [*n* = 1411 (35%)], and less frequently with endocarditis [*n* = 317 (8%)]. The median ICU length of stay and hospital length of stay were 6 days [2–14] and 21 days [13–38], respectively. In-hospital mortality was 27% (*n* = 1100) and 640 (16%) patients were secondarily transferred to rehabilitation care unit. Direct ICU group was significantly more severe but after adjustment for age, sex, comorbidities, organ failures on admission and admission from home, direct ICU admission was significantly associated with a lower mortality (Odds ratio 0.67 [0.56–0.80], *p* < 0.01). This corresponded to one death avoided for 11 PM directly admitted in ICU.

**Conclusions:**

Among patients with PM and sepsis, direct ICU admission was associated with lower mortality rates when compared to delayed admission.

**Supplementary Information:**

The online version contains supplementary material available at 10.1186/s13613-023-01239-1.

## Background

Pneumococcal meningitis remains the most common form of bacterial meningitis in adults in Europe and the United States [1–8] and is known to be associated with high mortality ranging from 15% to more than 33% [9–11] and morbidity, including hearing loss, in 10–40% of the patients. Although several advances were achieved in recent years to improve the prognosis of this disease, including the recommendations for steroids before or during initial antibiotic administration, and recommendations to reduce the delay in initial antibiotic administration, mortality and morbidity rates remain unsatisfactorily high, emphasizing the need to optimize treatment and immediate admission in intermediate or intensive care units (ICU). Current French guidelines recommend initial management of pneumococcal meningitis patients in ICU, while other guidelines such as European or American ones do not even mention any specific orientation [7, 12, 13]. However, evidence to support these recommendations is scarce [1]. Thus, only few data are available to assess the real burden of pneumococcal meningitis in the most severe cases, those with sepsis, i.e., with at least one organ failure and/or ICU admission. Most of these studies were retrospective, monocentric, with small number of patients, showing that most of the patients with pneumococcal meningitis admitted in ICU had most of the time consciousness impairment [14], including around 15% in comatose. None of these studies really focused on the hospital course of the patients, on their severity and the cost of care [15, 16]. In France, the medico-economic administrative hospital database called PMSI database (French acronym for “Programme de Médicalisation des Systèmes d'Information”) provides data on all the hospital stays in France of a total of 65 million people. Unlike other systems, PMSI includes mandatory calculation of SAPS II severity scores and daily monitoring of organ failure supplementation.

In this context, our aim was to describe the impact of direct ICU admission for pneumococcal meningitis with sepsis.

## Materials and methods

### Study design and setting

We analyzed all hospital stays in France from January 1st, 2011 to December 31st, 2020 of patients older than 18 years with codes associated with sepsis and pneumococcal meningitis based on the French medico-administrative (PMSI) database.

### Data source

The PMSI has been described elsewhere [17]. Briefly, it included the ICD-10 codes as well as the daily procedures and organ failure support performed for the patient’s care during hospitalization in all public and private hospitals by every resident in France. The Simplified Acute Physiology Score II (SAPS II) score was also systematically calculated for all patients admitted in ICU. The direct costs of each hospital stay were estimated using the base price of the Homogeneous Stay Group classification and the rates of daily add-on rates based on extra days and special procedures. Estimates of the total annual French population were provided by the French National Institute for Statistics and Economic Studies, a publicly available database of census data for the entire country [18].

### Definitions

We defined pneumococcal meningitis with at least one diagnostic code for bacterial meningitis and a code for pneumococcal, including pneumococcal pneumonia or pneumococcal septicemia. Sepsis was identified using ICD 10 and/or procedure codes according to the following definitions: (1) explicit definitions using the direct codes for sepsis [19, 20] or (2) implicit definitions using ICD-10 code for infection and a diagnostic code for organ failure for sepsis or a procedure code for the use of organ failure support for sepsis. The codes used for definitions are shown in the Additional file 1: Table S1. All the codes given during the entire hospital stays were considered. Septic shock was defined by an ICD-10 code for pneumococcal meningitis and an ICD-10 code for shock or the associated use of vasopressors. ICD-10 codes were also used to identify associated diagnoses, such as respiratory infection associated, cardiovascular infection, purpura, brainstem failure, seizure, coma, focal sign, hydrocephaly and vascular complication including ischemic stroke, transient ischemic stroke and cerebral venous thrombosis. A small group of patients were never admitted in ICU and were reported separately. Two groups of patients were defined according to care trajectories: direct ICU admission (“direct ICU”) and delayed ICU admission (“delayed ICU”). The “Direct ICU admission” subgroup included the patients directly admitted in ICU. “Delayed ICU admission” subgroup included the patients secondarily admitted in ICU. We explored the association between “direct ICU”, and clinically relevant outcomes including hospital mortality, ICU and hospital length of stay, hospital discharge characteristics and costs of care. Hospital characteristics (i.e., teaching versus non-teaching and private versus public hospital), SAPS II on admission, organ failures on admission and major clinical outcomes such as septic shock and death were assessed. We defined the mode of admission as: admission from home for patients who were directly hospitalized from home and admission from another hospital for patients hospitalized from another hospital. The different type of hospital was distinguished between public hospital including the group of universitary/regional hospital, the group of general/local hospital and other institutions, such as short-stay hospital or private hospital.

### Characteristics recorded

We considered a hospital stay from admission to death or discharge from acute care hospital, considering all potential transfers. For each hospital stay, we collected the following data: demographics (age, sex), hospital characteristics (teaching versus non-teaching and private versus public hospital), reason for admission (surgical versus medical), readmissions, wards to which patients were admitted (medical, intensive care, emergency) prior to ICU admission, patient comorbidities according to ICD-10 Charlson’s score [21], and severity illness on admission (organ failures and SAPS II only for the patients admitted in ICU). The direct costs of each hospital stay were estimated according to the Homogeneous Stay Group classification, which is associated with its financial counterpart, an amount set annually by the National Health Insurance. This rate is increased by daily supplements in the case of care in specialized units (intensive care units and intermediate care units). ICU and hospital length of stay, and outcomes including death and discharge to home were recorded. We included the specific care including neurosurgery and hospitalization in neurosurgical ICU with the procedure codes for intracranial pressure monitoring and external ventricular drain. The volume of first hospital was described according to admission rate for meningitis from 2011 to 2020 categorized by < 5 admission, between 5 and 10, between 10 and 20, between 20 and 50 and > 50 admissions. We defined patients with immediate sequelae requiring rehabilitation care as dependency equivalent to at least a mild disability (unable to carry out all previous activities, able to manage own affairs without assistance).

### Ethical aspects

According to the French regulatory system for personal and medical data and with the approval of the French National Commission for Data Protection and Liberties (CNIL), our institution was granted access to the PMSI database. We had access only to patients diagnosed with sepsis or septic shock according to our definition, and to pseudo-anonymized data. No nominative, sensitive or personal patient information was collected. The study does not involve human subjects and falls within the scope of the French Reference Methodology MR-004 according to the law 2016–41 of 26 January 2016 on the modernization of the French health system.

### Statistical analyses

Standard descriptive statistics were performed for the entire cohort and by year from 2011 to 2020. Data were summarized as frequency and percentage for categorical variables, and median and interquartile for continuous variables. Bivariate analyses were performed using Chi-square tests for categorical variables and Student *t* tests for continuous variables. Univariate and then multivariate logistic regression analyses were performed to identify the factors associated with death. Variables with a *p* value < 0.1 in the univariate analyses were retained in the multivariate analyses. Stepwise backward selection was performed in the multivariate analyses to select the final model.

Similarly, factors associated with death were examined in the subgroup of the patients without organ failure on first hospital admission. *P* values less than 0.05 were considered significant. Data were analyzed using SAS^®^ (version 9.4; SAS Institute, Cary, NC, USA) and R (version 3.4.0; R Core Team, Vienna, Austria).

## Results

### Main characteristics

From 2011 to 2020, 4052 patients were hospitalized in a French ICU for a first episode of pneumococcal meningitis with sepsis criteria. The characteristics of the excluded patients, who were not admitted in the ICU are presented in the Additional file 1: Table S2. Among the patients admitted in ICU, (*n* = 4052), mainly male [*n* = 2260 (56%)] with median age of 61 years [IQR 50–71] and a median Charlson index of 1 [0–3], 2006 patients (50%) were directly admitted (“Direct ICU” group) and 2046 patients (50%) were secondarily admitted (“Delayed ICU” group). Among them, median SAPS II on admission was 46 [33–62], 2173 (54%) had a neurological failure on admission, of which 2133 (53%) were in coma, 654 (16%) had brainstem failure, 488 (12%) had seizure and 779 (19%) had focal sign without coma. Their characteristics and their hospitalization details are shown in Table 1.

**Table 1:** Main characteristics of all patients and their hospitalization details with pneumococcal meningitis (n = 4052) directly admitted in ICU (*n* = 2006) or secondary admitted in ICU (*n* = 2046)

Variables [median IQR/*N* (%)]	Overall	Direct ICU admission	Delayed ICU admission	*p* value
	4052	2006	2046	.
Time from hospital to ICU admission	0 [0; 1]	0 [0; 0]	1 [0; 1]	< 0.01
Age, median [IQR]	61 [50–71]	60 [49–70]	62 [50–72]	< 0.01
Age > 65 years (%)	1647 (40.6)	765 (38.1)	882 (43.1)	< 0.01
Male (%)	2260 (55.8)	1141 (56.9)	1119 (54.7)	0.16
Comorbidities (%)				
Diabetes (%)	743 (18.3)	357 (17.8)	386 (18.9)	0.38
Cardiovascular disease (%)	550 (13.6)	252 (12.6)	298 (14.6)	0.06
Cancer (%)	496 (12.2)	166 (8.3)	330 (16.1)	< 0.01
Liver chronic disease (%)	416 (10.3)	185 (9.2)	231 (11.3)	0.03
Chronic kidney disease (%)	206 (5.1)	71 (3.5)	135 (6.6)	< 0.01
Charlson score, median [IQR]	1 [0–3]	1 [0–2]	1 [0–3]	< 0.01
Organ failure (OF) on admission				
Neurological failure on admission (%)	2173 (53.6)	1533 (76.4)	640 (31.3)	< 0.01
Respiratory failure on admission (%)	1626 (40.1)	1437 (71.6)	189 (9.2)	< 0.01
Cardiovascular failure on admission (%)	1462 (36.1)	1188 (59.2)	274 (13.4)	< 0.01
Renal failure on admission (%)	532 (13.1)	449 (22.4)	83 (4.1)	< 0.01
Hematological failure on admission (%)	198 (4.9)	165 (8.2)	33 (1.6)	< 0.01
Number of OF on admission, median [IQR]	1 [0–3]	2 [2, 3]	0 [0–1]	< 0.01
SAPS II during ICU stay, median [IQR]	46 [33–62]	48 [36–63]	44 [31–60]	< 0.01
Complications				
Coma (%)	2133 (52.6)	1117 (55.7)	1016 (49.7)	< 0.01
Focal sign without coma (%)	779 (19.2)	373 (18.6)	406 (19.8)	0.31
Brainstem failure (%)	654 (16.1)	356 (17.7)	298 (14.6)	< 0.01
Seizure (%)	488 (12)	231 (11.5)	257 (12.6)	0.31
Vascular complication (%)^a^	258 (6.4)	150 (7.5)	108 (5.3)	< 0.01
Purpura (%)	160 (3.9)	90 (4.5)	70 (3.4)	0.08
Hydrocephaly (%)	53 (1.3)	27 (1.3)	26 (1.3)	0.83
Neurosurgery (ICP and/or EVD) (%)	255 (6.3)	137 (6.8)	118 (5.8)	0.16
Associated infection				
Respiratory infection (%)	1411 (34.8)	712 (35.5)	699 (34.2)	0.37
Cardiovascular infection (%)	317 (7.8)	139 (6.9)	178 (8.7)	0.04
Category of first hospital				
Regional/universitary hospital (%)	1529 (37.7)	813 (40.5)	716 (35)	< 0.01
Local (%)	2079 (51.3)	1059 (52.8)	1020 (49.9)	.
Other (%)	444 (11)	134 (6.7)	310 (15.2)	.
Transfers				
Admission from home (%)	3909 (96.5)	1900 (94.7)	2009 (98.2)	< 0.01
Admission from another hospital (%)	139 (3.4)	106 (5.3)	33 (1.6)	< 0.01
Up category hospital (to regional hospital) (%)	479 (24.4)	171 (22.9)	308 (25.3)	0.23

The mode of admission was defined as: admission from home for patients who were directly hospitalized from home and admission from another hospital for patients hospitalized from another hospital. The ICD-10 codes used for definitions are displayed in the Additional file 1: Table S1

The different type of hospital was separated between public hospital including the group of universitary/regional hospital, the group of general/local hospital and other institutions like short hospitalization or private hospital

*ICU* intensive and intermediate care units, *ICP* intracranial pressure monitoring, *EVD* external ventricular drain, *IQR* interquartile

^a^Vascular complications included ischemic stroke, transient ischemic stroke and cerebral venous thrombosis

Pneumococcal meningitis with sepsis was frequently associated with pneumonia [*n* = 1411 (35%)], and less frequently with endocarditis [*n* = 317 (8%)]. The median ICU and hospital length of stays were 6 days [2–14] and 21 days [13–38], respectively. In-hospital mortality was 1100 (27%). A total of 640 (16%) were transferred to a rehabilitation care unit. The median cost of care was 39,556 € [IQR 22988–75,609 €]. Patient outcomes are shown in Table 2.

**Table 2:** Outcomes of patients with pneumococcal meningitis (*n* = 4052) directly admitted in ICU (*n* = 2006) or secondary admitted in ICU (*n* = 2046)

Variables [median IQR/N (%)]	Overall	Direct ICU admission	Delayed ICU admission	*p* value
**Population**	**4052**	**2006**	**2046**	.
**Outcomes**				
Hospital length of stay in days, median [IQR]	21 [13–38]	20.5 [13–37]	21 [13–39]	0.20
ICU length of stay in days, median [IQR]	6 [2–14]	7 [3–15]	5 [2–13]	< 0.01
Costs in euros, median [IQR]	39,556 [22988–75609]	36,441 [20780–64927]	43,490 [24357–83854]	< 0.01
Discharge home (%)	2033 (50.2)	1005 (50.1)	1028 (50.2)	0.29
Discharge hospital (%)	279 (6.9)	131 (6.5)	148 (7.2)	.
Discharge readaptation (%)	640 (15.8)	337 (16.8)	303 (14.8)	.
In-hospital death (%)	1100 (27.1)	533 (26.6)	567 (27.7)	.
Death at 1 year (%)	1183 (29.2)	561 (28)	622 (30.4)	0.09

*ICU* intensive and intermediate care units, *IQR* interquartile

### Comparison of direct ICU admission versus delayed ICU admission

Compared to the direct ICU group, the delayed ICU group had more comorbidities, with a higher Charlson index (*p* < 0.01), higher rates of cancer (330 (16%) versus 166 (8%), *p* < 0.01) and chronic kidney disease (135 (7%) versus 71 (4%), *p* < 0.01).

The Direct ICU group was significantly more severe on admission based on number of organ failures on admission (2 [2, 3] versus 0 [0–1], *p* < 0.01) and all types of organ failures on admission (*p* < 0.01). SAPS II during ICU stay was also significantly higher in the direct ICU group (48 [36–63] versus 44 [31–60], *p* < 0.01). No significant temporal trends in mortality due to pneumococcal meningitis were found from 2011 to 2020 (Additional file 1: Figure S1). Mortality was, respectively, up to 27% (*n* = 1100) during the hospital stay and 29% (n = 1183) at 1 year. The direct ICU group had a significantly longer ICU stay (7 [3–15] versus 5 [2–13], *p* < 0.01) but the total costs were significantly higher in the delayed ICU admission group (43,490 € [24357–83854] versus 36,441 € [20780–64927], *p* < 0.01).

**Fig. 1:**
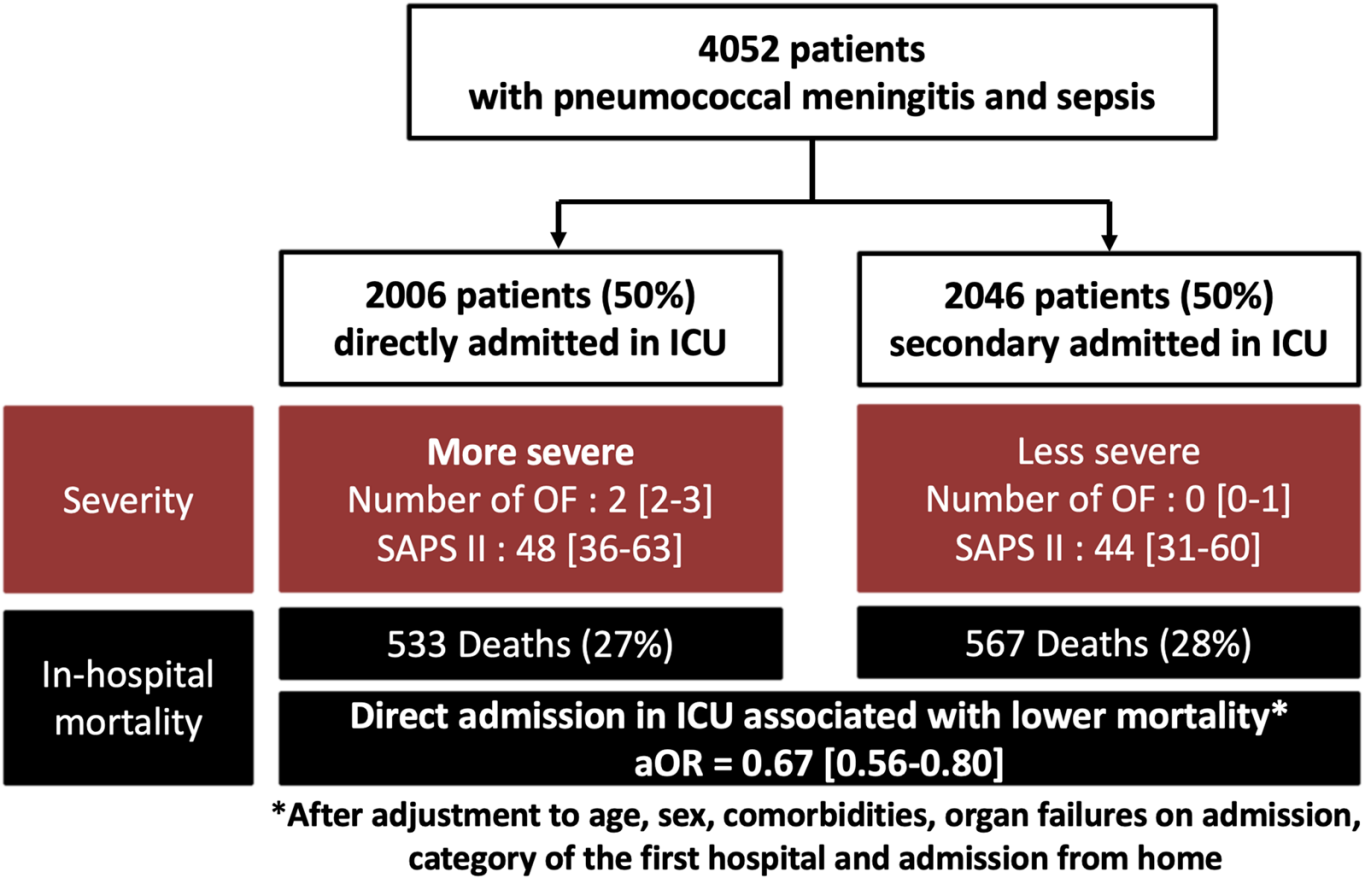
Flow chart. *ICU* intensive and intermediate care units, *OF *organ failures, *OR* odds ratio More severe: higher SAPS II, higher number of organ failure except neurological failure on admission, more septic shock on admission, more coma, more brainstem failure, more vascular complication mortality: in-hospital mortality

Age ≥ 65 years, organ failure on admission, associated respiratory infection, clinical signs of severity as brainstem failure, coma or purpura, category of the first hospital and direct ICU admission were associated with increased mortality in univariate analysis (Additional file 1: Table S3). After adjustment to age, sex, comorbidities, organ failure on admission, category of the first hospital, admission from home and associated pneumonia, direct ICU admission was associated with lower mortality (adjusted odds ratio (aOR) = 0.67 [0.56–0.80], *p* < 0.01). Univariate analysis is displayed in Additional file 1: Table S3, and multivariate analysis is displayed on Table 3.﻿ The Fig. 1 is a combination of a flow chart and a visual abstract, which provides a clear and concise overview of the study design and results. Sensitivity analysis after excluding the patients with organ failures on hospital admission, showed a non-significant difference on mortality associated with direct ICU admission (aOR = 0.76 [0.48–1.19], *p* = 0.22) (Additional file 1: Table S4).

**Table 3:** Multivariate analysis of factors associated with death among patients admitted in ICU for pneumococcal meningitis

Variable	Multivariate analysis		
	Odds Ratio	95% CI	*p* value
Age > 65 years	1.89	[1.63; 2.2]	< 0.01
Sex (Female)	0.79	[0.68–0.92]	< 0.01
Cancer	1.26	[1.02–1.56]	0.04
**Organ failures on admission**			
CV failure on admission	1.82	[1.52–2.17]	< 0.01
Renal failure on admission	2.31	[1.87–2.85]	< 0.01
Associated pneumonia	1.30	[1.12–1.51]	< 0.01
**Mode of admission**			
Admission from home	2.26	[1.38–3.7]	< 0.01
Direct ICU admission	0.67	[0.56–0.8]	< 0.01
**Category of 1st hospital**			
Regional/universitary hospital	1		
Local hospital	1.24	[1.06–1.46]	0.01
Other (private hospital, short hospitalization)	1.50	[1.17–1.92]	< 0.01

Volume admissions of hospital corresponding to the volume of admissions for meningitis. The ICD-10 codes used for definitions are displayed in the Additional file 1: Table S1. The variables tested in multivariate analysis were: the variables displayed here in the Table 3 and gender (female), cardiovascular comorbidities, chronic obstructive pulmonary disease, diabetes, chronic kidney disease, neurological, respiratory and hematological failures on admission, associated diagnosis of endocarditis. The mode of admission was defined as: admission from home for patients who were directly hospitalized from home and admission from another hospital for patients hospitalized from another hospital

*95% CI* 95% confidence interval, *CV* cardiovascular, *ICU* intensive and intermediate care units

## Discussion

To the best of our knowledge, this is the first national study to evaluate the outcomes of patients with pneumococcal meningitis and sepsis in French hospitals as a function of the course of hospitalization and to find that direct ICU admission of these patients is independently associated with lower mortality. In our cohort, we also confirm the severity of pneumococcal meningitis with significant in-hospital mortality (27%) and morbidity (16% were secondarily transferred to rehabilitation care unit).

These main results deserve several comments. We have shown that pneumococcal meningitis is still associated with a relatively high mortality and morbidity. In our study, morbidity was illustrated by the transfer to a rehabilitation care unit. We believed that transfer to a rehabilitation care unit was an important point on outcomes and sequelae for recovery from pneumococcal meningitis [22]. Previous studies found that pneumococcal meningitis was associated with poor outcomes as assessed by the modified Rankin Scale or mostly Glasgow Outcome Scale rating the disability at discharge from 34 to 45% [1, 4, 14]. In our study, the one-year mortality was 29%, but we did not have the details on long-term disability. The PMSI does not include home rehabilitation.

Then, regarding the impact of immediate ICU admission on mortality, many studies showed that a lower priority for ICU admission of septic patients without septic shock than patients with other indications for ICU admission was associated with significantly higher mortality [23–25]. We also found higher total costs in the delayed ICU admission group. The benefits of direct admission in the ICU may be due to early and adequate antibiotic treatment in the management of pneumococcal meningitis, including the use of corticosteroids [26–29], or better monitoring and prevention of secondary deterioration [30]. Inappropriate triage in the first place may also be important. As diagnoses were identified on the basis of the entire hospital stay and not on the basis of organ failure (CIM10 code) versus CCAM code, we could not be sure that the diagnosis of meningitis was made at hospital admission. We could not exclude heterogeneity in the general management of patients (i.e., ICU admission policy, appropriate treatment, etc.) between centers. However, center characteristics were included in the final model. A mixed model using center as an additional random-effect gave very similar results (data not shown). The predictors of sepsis severity at the front of triage could be useful but may lack specificity for pneumococcal meningitis on mortality and neurological outcomes [14, 31]. In our study, the patients admitted directly to ICU were more severe including pneumococcal meningitis severity criteria which is consistent with existing literature [32–34]. Due to this important confounding factor on mortality, we included the “direct ICU admission” variable in the multivariate analysis for adjustment despite the results of the univariate analysis. Other clinical signs also showed an independent risk factor for poor outcome, such as initial seizures, which we did not find in our study [35, 36].

Finally, the benefit of immediate ICU admission is not significant in patients without organ failures on hospital admission. Consequently, the patients with any organ failure should be the priority on direct ICU admission.

The main strength of our study was the size of the population, which included all patients in France with their hospital trajectories, and the most comprehensive adjustment for all available individual factors. The adjustment for many confounding factors (including the type of hospital and subgroup analysis) finally allowed us to conclude that direct admission was associated with lower mortality compared with delayed ICU admission, despite its severity. The description of the main characteristics from Table 1 showed that the most severe patients (with higher organ failures) were mostly directly admitted in the ICU. This could be explained by the initial appropriate triage of the more severe patients admitted directly to the ICU, who are more likely to have higher mortality. However, after adjusting for severity scores and organ failure on admission in the multivariate analysis (Table 3), we found a protective effect of direct ICU admission, meaning that for the same severity and organ failure, direct admission of pneumococcal meningitis patients with sepsis is independently associated with lower mortality (*p* < 0.01). The higher total costs in the delayed ICU admission (*p* < 0.01) also support the current guidelines to admit these patients with pneumococcal meningitis directly in ICU. To avoid immortality bias, we included patients at the beginning of their disease, i.e., at their first contact with the hospital. We chose to merge the two types of intermediate or intensive care units, because it was not possible to separate intermediate and intensive care units according to the units available by hospital, and guidelines recommend managing these patients in intermediate or intensive care units. Overall, our study suggests an insufficient compliance with the actual guidelines [7, 12, 13]. Indeed, half of the patients with pneumococcal meningitis and sepsis were not admitted in first place in ICU. Yet, this fact could be due to other factors such as another first associated diagnosis (e.g., pneumococcal pneumonia), a premortem misdiagnosis or early limitation of care including patients with more comorbidities who were not admitted directly in ICU. All of these factors could lead to a lack of adjuvant therapy as corticosteroids. This also led us to exclude patients who were not admitted in ICU [28, 37]. In our study, we could not evaluate when the ICU was full or if triage was done by telephone [38]. The mortality among patients who were not admitted in ICU units (*n* = 449) was similar to the patients admitted in ICU (*p* = 0.55), making the risk of an immortality bias unlikely.

Despite the important findings, our study has several limitations. First, it was a national French hospital, which may limit the external validity. However, it was a multicenter study and the large sample size gives some robustness to our results. Second, the retrospective nature of this investigation did not allow us to assess some parameters that could have been relevant, such as antibiotic therapy (delay, type, duration, etc.), but also adjuvant therapy, such as corticosteroids. Treatment details in the PMSI database were not available for most of the patients included in the study, while patients may not be treated promptly and appropriately by all physicians. Third, the study was based on codes that largely depend on the quality of the data. However, the PMSI database remained reliable in several studies [17, 39–42]. This method made it difficult to obtain important details, such as the exact timing of the onset of organ failure leading to ICU admission, which may be related to the cause of admission or ICU deterioration. Two different sepsis criteria may be used between 2011 and 2020, but differences between the two definitions of sepsis were limited by using sepsis definition based on ICD 10 and/or procedure codes specified in the methods. The lack of details such as biological outcomes may explain differences in prognosis, initial triage, long-term outcomes or potential sequelae. Finally, this study involved pneumococcal meningitis with sepsis and not a general population of pneumococcal meningitis patients.

## Conclusion

In France, from 2011 to 2020, most of pneumococcal meningitis with sepsis was admitted in ICU, directly or indirectly. Among patients with PM and sepsis, direct ICU admission was associated with lower mortality rates when compared to delayed admission, supporting actual guidelines which were insufficiently followed.

### Supplementary Information

**Additional file 1: Figure S1.** Incidence and mortality of pneumococcal meningitis over years. **Table S1.** International Classification of diseases, 10th revision (ICD-10), codes used for the identifications of infections, organ failures and comorbidities. **Table S2.** Comparison of patients not admitted to ICU/IntermCU versus the others. **Table S3.** Univariate analysis of factors associated with death among patients admitted in ICU for pneumococcal meningitis. **Table S4.** Risk factors for hospital death among patients without organ failure on hospital admission.

## Data Availability

Data available on request.
