# Supplementary material for: Impact of direct ICU admission of pneumococcal meningitis in France: a retrospective analysis of a French medico-administrative (PMSI) database
Source: Ann Intensive Care. 2024 Jan 27;14:15. doi: 10.1186/s13613-023-01239-1 (PMC10817881; doi:10.1186/s13613-023-01239-1)
Supplement: Supplementary file 1 — Additional file 1: Figure S1. Incidence and mortality of pneumococcal meningitis over years. Table S1. International Classification of diseases, 10th revision (ICD-10), codes used for the identifications of infections, organ failures and comorbidities. Table S2. Comparison of patients not admitted to ICU/IntermCU versus the others. Table S3. Univariate analysis of factors associated with death among patients admitted in ICU for pneumococcal meningitis. Table S4. Risk factors for hospital death among patients without organ failure on hospital admission. [file 13613_2023_1239_MOESM1_ESM.docx]

**Additional file materials**

**Impact of direct ICU admission of pneumococcal meningitis in France: a retrospective analysis of a French medico-administrative (PMSI) database**

Table of contents:

- Additional file Figure 1: Incidence and mortality of pneumococcal meningitis over years
- Additional file Table S1: International Classification of diseases, 10th revision (ICD-10), codes used for the identifications of infections, organ failures and comorbidities
- Additional file Table S2: Comparison of patients not admitted to ICU/IntermCU versus the others
- Additional file Table S3: Univariate analysis of factors associated with death among patients admitted in ICU for pneumococcal meningitis
- Additional file Table S4: Risk factors for hospital death among patients without organ failure on hospital admission

Additional file **1: Figure 1: Incidence and mortality of pneumococcal meningitis over years**


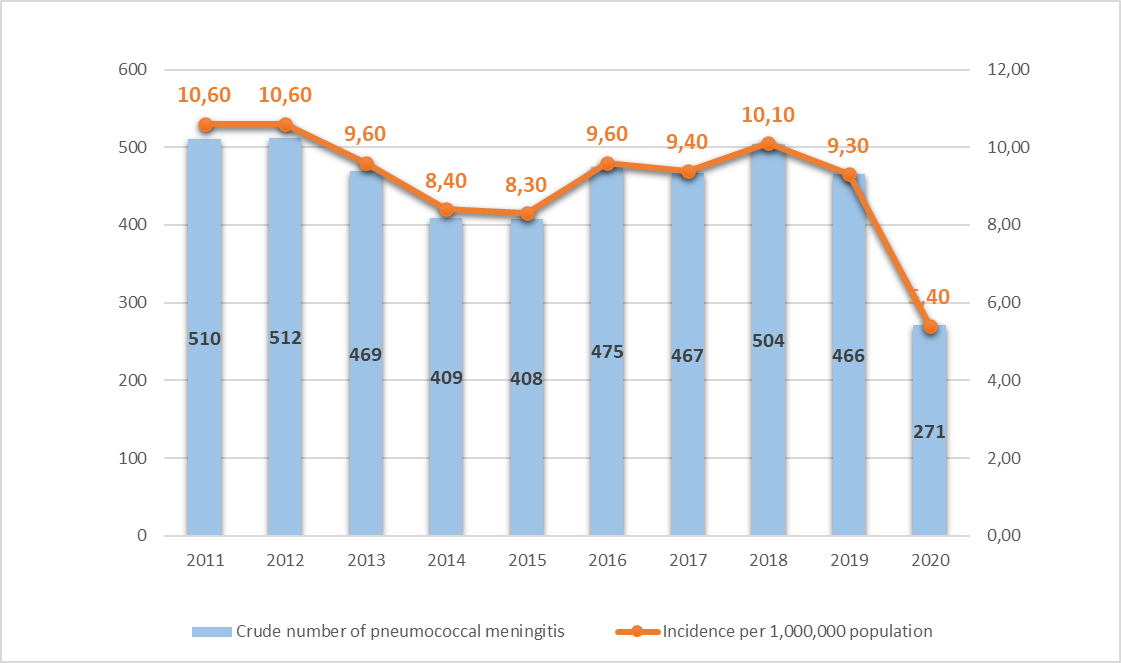


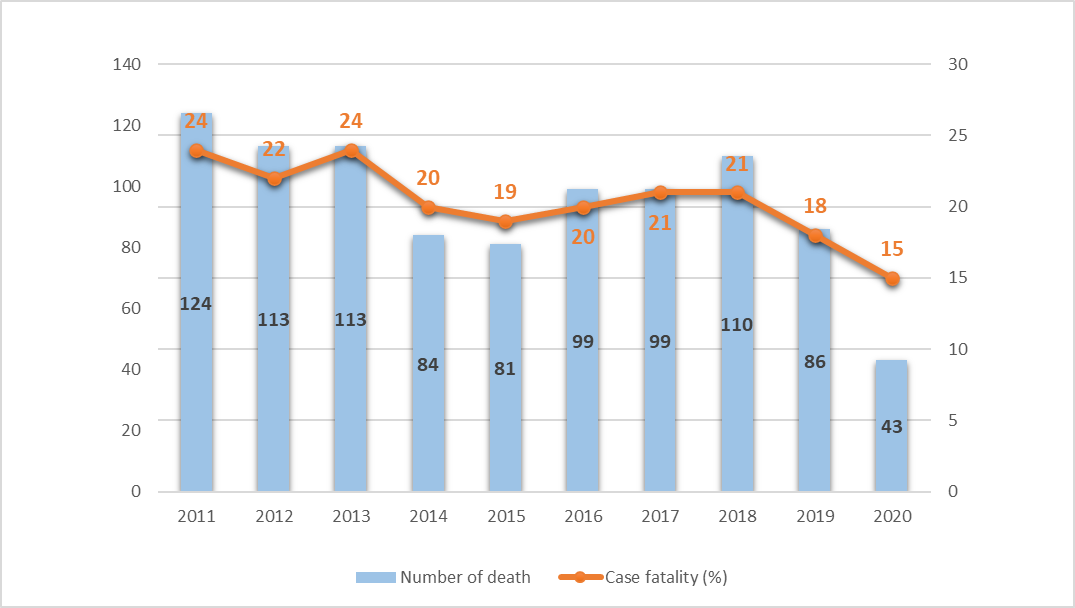


Additional file **1: Table S1: International Classification of diseases, 10th revision (ICD-10), codes used for the identifications of infections, organ failures and comorbidities**

| Label | ICD-10 code |
| --- | --- |
| Infection | A000-B99, G000, G00, G001, G002, G003, G008, G009, G01, G02, G020, G021, G028, G04, G042, G049, G050, G051, G052, G06, G060, G061, G062, G08, H030, H031, H054, H061, H130, H131, H132, H133, H138, H600, H601, H602, H603, H620, H621, H622, H623, H670, H671, H68, H680, H750, H758, I010, I011, I012, I090, I091, I092, I30, I301, I309, I320, I321, I33, I330, I339, I38, I39, I398, I40, I400, I401, I408, I409, I41, I410, I411, I412, I418, I430, I514, I681, I891, I980, I981, J00, J01X, J02X, J03X, J04X, J05X, J06X, J09, J10X, J11X, J12X, J13, J14, J15X, J16X, J17X, J180, J181, J182, J188, J189x, J18X, J20, J200, J201, J202, J203, J204, J205, J206, J207, J208, J209, J21, J210, J211, J218, J219, J22, J36, J390, J391, J40, J440, J85, J850, J851, J852, J853, J86, J860, J869, J953, J961, J982, J983, K046, K047, K050, K052, K103, K113, K122, K230, K231, K238, K2380, K2381, K35, K350, K351, K352, K353, K358, K359, K36, K37, K431, K434, K437, K441, K451, K461, K550, K551, K558, K559, K570, K572, K574, K578, K61, K61, K610, K610, K611, K611, K612, K612, K613, K613, K614, K614, K630, K631, K65, K650, K658, K659, K66, K67, K670, K671, K672, K673, K678, K750, K751, K764, K770, K800, K801, K803, K804, K81, K810, K818, K819, K821, K822, K823, K830, K831, K832, K833, K8700, K930, K93820, K93840, L01, L010, L011, L02, L020, L021, L022, L023, L024, L028, L029, L03, L030, L031, L032, L033, L038, L039, L04, L040, L041, L042, L043, L048, L049, L05, L050, L059, L08, L080, L081, L088, L089, L303, M00, M000, M0000, M0001, M0002, M0003, M0004, M0005, M0006, M0007, M0008, M0009, M001, M0010, M0011, M0012, M0013, M0014, M0015, M0016, M0017, M0018, M0019, M002, M0020, M0021, M0022, M0023, M0024, M0025, M0026, M0027, M0028, M0029, M008, M0080, M0081, M0082, M0083, M0084, M0085, M0086, M0087, M0088, M0089, M009, M0090, M0091, M0092, M0093, M0094, M0095, M0096, M0097, M0098, M0099, M01, M010, M0100, M0101, M0102, M0103, M0104, M0105, M0106, M0107, M0108, M0109, M011, M0110, M0111, M0112, M0113, M0114, M0115, M0116, M0117, M0118, M0119, M012, M0120, M0121, M0122, M0123, M0124, M0125, M0126, M0127, M0128, M0129, M013, M0130, M0131, M0132, M0133, M0134, M0135, M0136, M0137, M0138, M0139, M014, M0140, M0141, M0142, M0143, M0144, M0145, M0146, M0147, M0148, M0149, M015, M0150, M0151, M0152, M0153, M0154, M0155, M0156, M0157, M0158, M0159, M016, M0160, M0161, M0162, M0163, M0164, M0165, M0166, M0167, M0168, M0169, M018, M0180, M0181, M0182, M0183, M0184, M0185, M0186, M0187, M0188, M0189, M462, M4620, M4621, M4622, M4623, M4624, M4625, M4626, M4627, M4628, M4629, M463, M4630, M4632, M4633, M4634, M4635, M4636, M4637, M4638, M4639, M464, M4640, M4642, M4643, M4644, M4645, M4646, M4647, M4648, M4649, M465, M4650, M4651, M4652, M4653, M4654, M4655, M4656, M4657, M4658, M4659, M490, M4900, M4901, M4902, M4903, M4904, M4905, M4906, M4907, M4908, M4909, M491, M4910, M4911, M4912, M4913, M4914, M4915, M4916, M4917, M4918, M4919, M492, M4920, M4921, M4922, M4923, M4924, M4925, M4926, M4927, M4928, M4929, M493, M4930, M4931, M4932, M4933, M4934, M4935, M4936, M4937, M4938, M4939, M60, M600, M6000, M6001, M6002, M6003, M6004, M6005, M6006, M6007, M6008, M6009, M630, M631, M632, M65, M650, M6500, M6501, M6502, M6503, M6504, M6505, M6506, M6507, M6508, M6509, M651, M6510, M6511, M6512, M6513, M6514, M6515, M6516, M6517, M6518, M6519, M711, M7110, M7111, M7112, M7113, M7114, M7115, M7116, M7117, M7118, M7119, M726, M7260, M7261, M7262, M7263, M7264, M7265, M7266, M7267, M7268, M7269, M730, M7300, M7301, M7302, M7303, M7304, M7305, M7306, M7307, M7308, M7309, M731, M7310, M7311, M7312, M7313, M7314, M7315, M7316, M7317, M7318, M7319, M86, M860, M8600, M8601, M8602, M8603, M8604, M8605, M8606, M8607, M8608, M8609, M861, M8610, M8611, M8612, M8613, M8614, M8615, M8616, M8617, M8618, M8619, M862, M8620, M8621, M8622, M8623, M8624, M8625, M8626, M8627, M8628, M8629, M864, M8640, M8641, M8642, M8643, M8644, M8645, M8646, M8647, M8648, M8649, M865, M8650, M8651, M8652, M8653, M8654, M8655, M8656, M8657, M8658, M8659, M868, M8680, M8681, M8682, M8683, M8684, M8685, M8686, M8687, M8688, M8689, M869, M8690, M8691, M8692, M8693, M8694, M8695, M8696, M8697, M8698, M8699, M90, M900, M9000, M9001, M9002, M9003, M9004, M9005, M9006, M9007, M9008, M9009, M901, M9010, M9011, M9012, M9013, M9014, M9015, M9016, M9017, M9018, M9019, M902, M9020, M9021, M9022, M9023, M9024, M9025, M9026, M9027, M9028, M9029, N10, N110, N111, N12, N136, N151, N160, N290, N291, N30, N300, N309, N330, N34, N340, N342, N369, N390, N410, N411, N412, N413, N419, N431, N45, N450, N459, N70, N700, N709, N710, N719, N72, N73, N730, N731, N732, N733, N734, N735, N739, N740, N741, N742, N743, N744, N751, N760, N761, N762, N763, N764, N770, N771, O040, O045, O050, O080, O23, O230, O231, O232, O233, O234, O235, O239, O411, O740, O752, O753, O85, O86, O860, O861, O862, O863, O864, O868, O883, O91, O910, O911, O98, O980, O981, O982, O983, O984, O985, O986, O987, O988, O989, R02, R50, R500, R501, R508, R509, R55, R568, R57, R572, R578, R579, R619, R65, R650, R651, R659, R680, R75, R827, R835, R845, R855, R865, R875, T793, T802, T814, T826, T827, T835, T836, T845, T846, T847, T857, T874, T880, U04, U049 |
| Type of germs | |
| Fungal infection | B35, B350, B351, B352, B353, B354, B355, B356, B358, B359, B36, B360, B361, B362, B363, B368, B369, B37, B370, B371, B372, B373, B373+, B374, B375, B375+, B376, B376+, B377, B378, B379, B38, B380, B381, B382, B383, B384, B384+, B387, B388, B389, B39, B390, B391, B392, B393, B394, B395, B399, B40, B400, B401, B402, B403, B407, B408, B409, B41, B410, B417, B418, B419, B42, B420, B420+, B421, B427, B428, B429, B43, B430, B431, B432, B438, B439, B44, B440, B441, B442, B447, B448, B449, B45, B450, B451, B452, B453, B457, B458, B459, B46, B460, B461, B462, B463, B464, B465, B468, B469, B47, B470, B471, B479, B48, B480, B481, B482, B483, B484, B487, B488, B49, C840, J67, L22, P375, B35 |
| Parasitic infection | A06, A060, A061, A062, A063, A064, A065+, A066+, A067, A068, A069, A07, A070, A071, A072, A073, A078, A079, J173, K231, O986, O988, A065, A590, A066, A081, A598, A599, B350, B351, B352, B353, B354, B355, B356, B358, B359, B360, B361, B362, B363, B500, B508, B509, B510, B518, B519, B520, B528, B529, B530, B531, B538, B54, B550, B551, B552, B559, B560, B561, B569, B570, B571, B572, B573, B574, B575, B580, B581, B582, B583, B588, B589, B59, B600, B601, B602, B608, B6080, B6088, B64, B850, B851, B852, B853, B854, B86, B870, B871, B872, B873, B874, B878, B879, B880, B881, B882, B883, B888, B889, B89, A59, A06, B50, B52, B51, B53, B55, B56, B57, B570+, B58, B580+, B581+, B582+, B583+, B59+, B60, B65, B650, B651, B652, B653, B658, B659, B66, B660, B661, B662, B663, B664, B665, B668, B669, B67, B670, B671, B672, B673, B674, B675, B676, B677, B678, B679, B68, B680, B681, B689, B69, B690, B691, B698, B699, B70, B700, B701, B71, B710, B711, B718, B719, B72, B73, B74, B740, B741, B742, B743, B744, B748, B749, B75, B76, B760, B761, B768, B769, B77, B770, B778, B779, B78, B780, B781, B787, B789, B79, B80, B81, B810, B811, B812, B813, B814, B818, B82, B820, B829, B83, B830, B831, B832, B833, B834, B838, B839, B85, B87, B88 |
| Virus infection | A080, A081, A082, A083, A084, A60, A600, A601, A609, A630, A80, A800, A801, A802, A803, A804, A809, A81, A810, A811, A812, A818, A819, A82, A820, A821, A829, A83, A830, A831, A832, A833, A834, A835, A836, A838, A839, A84, A840, A841, A848, A849, A85, A850, A850+, A851, A851+, A852, A858, A86, A87, A870, A870+, A871, A871+, A872, A878, A879, A88, A880, A881, A888, A89, A90, A91, A92, A920, A921, A922, A923, A924, A928, A929, A93, A930, A931, A932, A938, A94, A95, A950, A951, A959, A96, A960, A961, A962, A968, A969, A980, A981, A982, A983, A984, A985, A988, A99, B000, B001, B002, B003, B004, B005, B007, B008, B009, B010, B011, B012, B018, B019, B020, B021, B022, B023, B027, B028, B029, B03, B04, B050, B051, B052, B053, B054, B058, B059, B060, B068, B069, B07, B080, B081, B082, B083, B084, B085, B088, B09, B150, B159, B160, B161, B162, B169, B17.9, B170, B171, B172, B178, B180, B181, B182, B188, B189, B190, B199, B200, B201, B202, B203, B204, B205, B206, B207, B208, B209, B210, B211, B212, B213, B217, B218, B219, B220, B221, B222, B227, B230, B231, B232, B238, B24, B24+0, B24+1, B24+9, B250, B251, B252, B258, B259, B260, B261, B262, B263, B268, B269, B270, B271, B278, B279, B300, B301, B302, B303, B308, B309, B330, B331, B332, B333, B338, B340, B341, B342, B343, B344, B348, B349, B91, B941, B942, B970, B971, B972, B973, B974, B975, B976, B977, B978, G020, G051, I411, J09, J10, J100, J101, J108, J11, J110, J111, J118, J12, J120, J121, J122, J123, J128, J129, J171, J203, J204, J205, J206, J207, J210, J211, K2380, K8700, K93820, K93840, M014, M0140, M0141, M0142, M0143, M0144, M0145, M0146, M0147, M0148, M0149, M015, M0150, M0151, M0152, M0153, M0154, M0155, M0156, M0157, M0158, M0159, O984, O985, O987 |
| Mycobacteria | A15, A150, A151, A152, A153, A154, A155, A156, A157, A158, A159, A16, A160, A161, A162, A163, A164, A165, A166, A167, A168, A169, A17+, A170, A170+, A171, A171+, A178, A178+, A179, A179+, A18, A180, A180+, A181, A182, A183, A184, A185, A186, A187, A187+, A188, A19, A190, A191, A192, A198, A199, A279, A30, A300, A301, A302, A303, A304, A305, A308, A309, A31, A310, A311, A318, A319, A65, B900, B901, B902, B908, B909, B92, K230, K673, M011, M0110, M0111, M0112, M0113, M0115, M0116, M0117, M0118, M0119, M490, M4900, M4901, M4902, M4903, M4904, M4905, M4906, M4907, M4908, M4909, M900, M9000, M9001, M9002, M9003, M9004, M9005, M9006, M9007, M9008, M9009, N330, N740, N741, O980, M0114, M0114 |
| Anaerobic germs | A047, A051, A052, A054, A33, A414, A42, A420, A421, A422, A427, A428, A429, B967, A047 |
| Intracellular germs | A34, A35, A55, A56, A560, A561, A562, A563, A564, A568, A70, A71, A710, A711, A719, A74, A740+, A748, A749, A75, A750, A751, A752, A753, A759, A77, A770, A771, A772, A773, A778, A779, A78, A79, A790, A791, A798, A799, J160, K670 |
| Gram negatif bacilli | A00, A000, A001, A009, A01, A010, A011, A012, A013, A014, A02, A020, A021, A022, A022+, A028, A029, A03, A030, A031, A032, A033, A038, A039, A04, A040, A041, A042, A043, A044, A045, A046, A053, A20, A200, A201, A202, A203, A207, A208, A209, A21, A210, A211, A212, A213, A217, A218, A219, A23, A230, A231, A232, A233, A238, A239, A24, A240, A241, A242, A243, A244, A25, A250, A251, A259, A26, A260, A267, A268, A269, A280, A281, A282, A37, A370, A371, A378, A379, A413, A415, A430, A438, A44, A440, A441, A448, A449, A480, A481, A482, A484, A492, A57, B961, B962, B963, B964, B965, B966, B9680, B9681, B980, G000, J14, J150, J151, J155, J156, J201, M491, M4910, M4911, M4912, M4913, M4914, M4915, M4916, M4917, M4918, M4919, M492, M4920, M4921, M4922, M4923, M4924, M4925, M4926, M4927, M4928, M4929 |
| Gram positive cocci | A050, A38, A40, A400, A401, A402, A403, A408, A409, A410, A411, A412, A46, A483, A490, A491, A544, A549, B95, B950, B951, B952, B953, B954, B955, B956, B957, B958, G001, G002, G003, J020, J030, J13, J152, J153, J154, J202, L01, L010, L011, M000, M0000, M0001, M0002, M0003, M0004, M0005, M0006, M0007, M0008, M0009, M001, M0010, M0011, M0012, M0013, M0014, M0015, M0016, M0017, M0018, M0019, M002, M0020, M0021, M0022, M0023, M0024, M0025, M0026, M0027, M0028, M0029, M009, M0090, M0091, M0092, M0093, M0094, M0095, M0096, M0097, M0098, M0099, M463, M4630, M4632, M4633, M4634, M4635, M4636, M4637, M4638, M4639, A050 |
| Source of infection | |
| Neurological infection | G00, G000, G001, G002, G003, G008, G009, G01, G02, G020, G021, G028, G03, G030, G031, G032, G038, G039, G04, G040, G041, G042, G048, G049, G05, G050, G051, G052, G058, G06, G060, G061, G062, G07, G08, G09, I681 |
| Cardio vasucular infection | I011, I012, I010, I090, I091, I092, I30, I301, I309, I320, I321, I33, I330, I339, I38, I39, I398, I40, I400, I401, I408, I409, I41, I410, I411, I412, I418, I430, I514, I891, I980, I981 |
| Upper respiratory track infection | J00, J01, J010, J011, J012, J013, J014, J018, J019, J02, J020, J028, J029, J03, J030, J038, J039, J04, J040, J041, J042, J05, J050, J051, J06, J060, J068, J069, J36, J390, J391 |
| Lower respiratory track infection | J09, J10, J100, J101, J108, J11, J110, J111, J118, J12, J120, J121, J122, J123, J128, J129, J13, J14, J15, J150, J151, J152, J153, J154, J155, J156, J157, J158, J159, J16, J160, J168, J17, J170, J171, J172, J173, J178, J18, J180, J181, J182, J188, J189, J20, J200, J201, J202, J203, J204, J205, J206, J207, J208, J209, J21, J210, J211, J218, J219, J22, J40, J440, J85, J850, J851, J852, J853, J86, J860, J869, J953, J961, J982, J983 |
| Abdominal infection | A090, A099, K047, K050, K052, K103, K113, K122, K230, K231, K238, K2380, K2381, K35, K350, K351, K352, K353, K358, K359, K36, K37, K431, K434, K437, K441, K451, K461, K550, K551, K558, K559, K570, K572, K574, K578, K61, K610, K611, K612, K613, K614, K630, K631, K65, K650, K658, K659, K66, K67, K670, K671, K672, K673, K678, K750, K751, K764, K770, K800, K801, K803, K804, K81, K810, K811, K818, K819, K821, K822, K823, K83, K830, K832, K833, K8700, K930, K93820, K93840, K046, K831 |
| Skin infection | L01, L010, L011, L02, L020, L021, L022, L023, L024, L028, L029, L03, L030, L031, L032, L033, L038, L039, L04, L040, L041, L042, L043, L048, L049, L05, L050, L059, L08, L080, L081, L088, L089, L303 |
| Osteoarticular infection | M00, M000, M0000, M0001, M0002, M0003, M0004, M0005, M0006, M0007, M0008, M0009, M001, M0010, M0011, M0012, M0013, M0014, M0015, M0016, M'0017, M0018, M0019, M002, M0020, M0021, M0022, M0023, M0024, M0025, M0026, M0027, M0028, M0029, M008, M0080, M0081, M0082, M0083, M0084, M0085, M0086, M0087, M0088, M0089, M009, M0090, M0091, M0092, M0093, M0094, M0095, M0096, M0097, M0098, M0099, M01, M010, M0100, M0101, M0102, M0103, M0104, M0105, M0106, M0107, M0108, M0109, M011, M0110, M0111, M0112, M0113, M0114, M0115, M0116, M0117, M0118, M0119, M012, M0120, M0121, M0122, M0123, M0124, M0125, M0126, M0127, M0128, M0129, M013, M0130, M0131, M0132, M0133, M0134, M0135, M0136, M0137, M0138, M0139, M014, M0140, M0141, M0142, M0143, M0144, M0145, M0146, M0147, M0148, M0149, M015, M0150, M0151, M0152, M0153, M0154, M0155, M0156, M0157, M0158, M0159, M016, M0160, M0161, M0162, M0163, M0164, M0165, M0166, M0167, M0168, M0169, M018, M0180, M0181, M0182, M0183, M0184, M0185, M0186, M0187, M0188, M0189, M462, M4620, M4621, M4622, M4623, M4624, M4625, M4626, M4627, M4628, M4629, M463, M4630, M4632, M4633, M4634, M4635, M4636, M4637, M4638, M4639, M464, M4640, M4642, M4643, M4644, M4645, M4646, M4647, M4648, M4649, M465, M4650, M4651, M4652, M4653, M4654, M4655, M4656, M4657, M4658, M4659, M490, M4900, M4901, M4902, M4903, M4904, M4905, M4906, M4907, M4908, M4909, M491, M4910, 'M4911, M4912, M4913, M4914, M4915, M4916, M4917, M4918, M4919, M492, M4920, M4921, M4922, M4923, M4924, M4925, M4926, M4927, M4928, M4929, M493, M4930, M4931, M4932, M4933, M4934, M4935, M4936, M4937, M4938, M4939, M60, M600, M6000, M6001, M6002, M6003, M6004, M6005, M6006, M6007, M6008, M6009, M630, M631, M632, M65, M650, M6500, M6501, M6502, M6503, M6504, M6505 |
| Urological infection | N10, N080, N110, N111, N12, N136, N151, N160, N290, N291, N30, N300, N301, N302, N303, N304, N308, N309, N33, N330, N338, N34, N340, N341, N342, N343, N369, N37, N370, N378, N390, N41, N410, N411, N412, N413, N418, N419, N431, N45, N450, N459, N512 , N70, N700, N701, N709, N71, N710, N711, N719, N72, N73, N730, N731, N732, N733, N734, N735, N736, N738, N739, N74, N740, N741, N742, N743, N744, N751, N760, N761, N762, N763, N764, N770, N771 |
| Septicemia | A021,A227,A267,A327,A40,A400,A401,A402,A403,A408,A409,A41,A410,A411,A412,A413,A414,A415,A418,A419,A427,A548,T802,T880,B377 |
| Organ failures | |
| Cardiovascular organ failure | I95, E86, E872, I951, I958, I959, I981 R55, R571, R65, R651, R659 |
| Hematological organ failure | D65, D689, D695, D696, D762 |
| Liver organ failure | R17, K72, K720, K729 |
| Neurological organ failure | F05, F050, F058, F059, F079, F09, G934, R40, R400, R401, R4018, R402, R4028, R410, R451 |
| Renal organ failure | R392, R34, N17, N170, N171, N172, N178, N179, N19 |
| Respiratory organ failure | J80, J951, J952, J96, J960, J9600, J9601, J9609, J969, J9690, J9691, J9699, R092, R230 |
| Septic shock | R572, R578, R579 |
| Comorbidities according to an adaptation of the Charlson score | |
| Myocardial infarction | I20, I200, I201, I208, I209, I21, I210, I211, I212, I213, I214, I219, I22, I220, I221, I228, I229, I23, I230, I231, I232, I233, I234, I235, I236, I238, 'I24, I240, I241, I248, I249, I25, I250, I251, I252, I253, I254, I255, I256, I258, I259 |
| Congestive heart failure | I420, I421, I422, I423, I424, I425, I426, I427, I428, I429, 'I430, I431, I432, I438, I099, I110, I130, I132, I255, P290, I50, I500, I501, I509 |
| Peripheral vascular disease | I700, I701, I702, I708, I709, I7000, I7001, I7020, I7021, I7080, I7081, I7090, I7091, I71, I710, I711, I712, I713, I714, I715, I716, I718, I719, K551, K558, K559, Z958, Z959, I72, I720, I721, I722, I723, I724, I728, I729, I73, I730, I731, I738, I739, I77, I770, I771, I772, I773, I774, I775, I776, I778, I779, I78, I79, I790, I791, I792, I798 |
| Cerebrovascular disease | I60, I600, I601, I602, I603, I604, I605, I606, I607, I608, I609, 'I61, I610, I611, I612, I613, I614, I615, I616, I618, I619, I62, I620, I621, I629, I63, I630, I631, I632, I633, I634, I635, I636, I638, I639, I64, I65, I650, I651, I652, I653, I658, I659, I66, I660, I661, I662, I663, I664, I668, I669, I67, I670, I671, I672, I673, I674, I675, I676, I677, I678, I679, I68, I680, I681, I682, I688, I69, I690, I691, I692, I693, I694, I698, H340, H341, H342, H348, H349, H34, G45, G450, G451, G452, G453, 'G454, G458, G459, G46, G460, G461, G462, G463, G464, G465, G466, G467, G468, I725, I726, I720 |
| Dementia | F001, F00, F000, F001*, F002, F009, F01, F010, F011, F012, F013, F018, F019, F02, F020, F021, F022, F023, F024, F028, F03, F04, F051, G30, G300, G301, 'G301, G308, G309, G310, G311, G312, G318, G319, G32, G320, G328 |
| Chronic pulmonary disease | J40, I278, I279, J40, J41, J410, J411, J418, J42, J43, J430, J431, J432, J438, J439, J44, J440, J441, J448, J449, J450, J451, J458, J459, 'J46, J47, J60, J61, J620, J628, J630, J631, J632, J633, J634, J635, J638, J64, J65, J660, J661, J662, J668, J670, J671, J672, J673, J674, J675, J676, J677, J678, J679, J684, J701, J703, J96, J9610, J96100, J96101, J9611, J96110, J96111, J9619, J96190, J96191, J9690, J9691, J9699 |
| Rheumatic disease | J990, M050, M0500, M0501, M0502, M0503, M0504, M0505, M0506, M0507, M0508, M0509, M051, M0510, M0511, M0512, M0513, M0514, M0515, M0516, M0517, M0518, M0519, M052, M0520, M0521, M0522, M0523, M0524, M0525, M0526, M0527, M0528, M0529, M053, M0530, M0531, M0532, M0533, M0534, M0535, M0536, M0537, M0538, M0539, M058, M0580, M0581, M0582, M0583, M0584, M0585, M0586, M0587, M0588, M0589, M059, M0590, M0591, M0592, M0593, M0594, M0595, M0596, M0597, M0598, M0599, M060, M0600, M0601, M0602, M0603, M0604, M0605, M0606, M0607, M0608, M0609, M061, M0610, M0611, M0612, M0613, M0614, M0615, M0616, M0617, M0618, M0619, M062, M0620, M0621, M0622, M0623, M0624, M0625, M0626, M0627, M0628, M0629, M063, M0630, M0631, M0632, M0633, M0634, M0635, M0636, M0637, M0638, M0639, M064, M0640, M0641, M0642, M0643, M0644, M0645, M0646, M0647, M0648, M0649, M068, M0680, M0681, M0682, M0683, M0684, M0685, M0686, M0687, M0688, M0689, M069, M0690, M0691, M0692, M0693, M0694, M0695, M0696, M0697, M0698, M0699, M30, M300, M301, M302, M303, M308, M31, M310, M311, M312, M313, M314, M315, M316, M317, M318, M319, M32, M320, M321, M321+, M328, M329, M33, M330, M331, M332, M339, M34, M340, M341, M342, M348, M349, M35, M350, M351, M352, M353, M354, M355, M356, M357, M358, M359, M36, M360, M361, M362, M363, M364, M368, M42, M420, M421, M429, M60, M600, M601, M602, M608, M609, M61, M610, M611, M612, M613, M614, M615, M619, M633, M638 |
| Peptic ulcer disease | K250, K251, K252, K253, K254, K255, K256, K257, K259, K260, K261, K262, K263, K264, K265, K266, K267, K269, K270, K271, 'K272, K273, K274, K275, K276, K277, K279, K280, K281, K282, K283, K284, K285, K286, K287, K289, K221, K223, K226, K25, 'K26, K27, K28, K29, K290, K291, K292, K293, K294, K295, K296, K297, K298, K299 |
| Mild liver disease | B18, B180, B181, B182, B188, B189, K70, K700, K701, K702, K703, K704, K709, K71, K710, K711, K712, K713, K714, K715, K716, K717, K718, K719, K72, K720, K721, K729, K73, K730, K731, K732, K738, K739, K75, K750, K751, K752, K753, K754, K758, K759, K76, K760, K761, K762, K763, K764, K765, K766, K767, K768, K769, K77, K770, K778, Z944 |
| Diabetes without chronic complication | E10, E100, E101, E106, E108, E109, E11, E110, E1100, E1108, E111, E1110, E1118, E116, E118, E119, E1190, E1198, E12, E120, E121, E126, E128, E129, E13, E130, E131, E136, E138, E139, E14, E140, E141, E146, E148, E149, O24, O240, O241, O242, O243, O244, O249 |
| Diabetes with chronic complication | E102, E102+, E103, E103+, E104, E104+, E105, E106, E107, E108, E112, E112+, E1120, E1128, E113, E113+, E1130, E1138, E114, E114+, E1140, E1148, E115, E1150, E1158, E116, E1160, E1168, E117, E1170, E1178, E118, E1180, E1188, E122, E122+, E123, E123+, E124, E124+, E125, E126, E127, E128, E132, E132+, E133, E133+, E134, E134+, E135, E136, E137, E138, E142, E142+, E143, E143+, E144, E144+, E145, E146, E147, E148 |
| Hemiplegia or paraplegia | G041, G114, G80, G800, G801, G802, G803, G804, G808, G809, G81, G810, G811, G819, G82, G820, G821, G822, G823, G824, G825, G83, G830, G831, G832, G833, G834, G838, G838+0, G838+8, G839 |
| Chronic kidney disease | N030, I120, I129, I130, I131, I132, I139, I150, I151, N00, N01, N02, N03, N031, N0310, N0319, N032, N033, N0330, N0339, N034, N035, N036, N037, N038, N0380, N0389, N039, N04, N040, N0400, N0409, N041, N042, N043, N044, N045, N046, N047, N048, N049, N05, N050, N051, N052, N053, N054, N055, N056, N057, N058, N059, N10, N11, N110, N111, N118, N119, N12, N13, N130, N131, N132, N133, N134, N135, N136, N137, N138, N139, N14, N140, N141, N142, N143, N144, N15, N150, N151, N158, N159, N16, N160, N161, N162, N163, N164, N165, N168, N17, N170, N171, N172, N178, N179, N18, N180, N181, N182, N183, N184, N185, N188, N189 |
| Any malignancy | C000, C00, C000, C001, C002, C003, C004, C005, C006, C008, C009, C01, C02, C020, C021, C022, C023, C024, C028, C029, C03, C030, C031, C039, C04, C040, C041, C048, C049, C05, C050, C051, C052, C058, C059, C06, C060, C061, C062, C068, C069, C07, C08, C080, C081, C088, C089, C09, C090, C091, C098, C099, C10, C100, C101, C102, C103, C104, C108, C109, C11, C110, C111, C112, C113, C118, C119, C12, C13, C130, C131, C132, C138, C139, C14, C140, C142, C148, C15, C150, C151, C152, C153, C154, C155, C158, C159, C16, C160, C161, C162, C163, C164, C165, C166, C168, C169, C17, C170, C171, C172, C173, C178, C179, C18, C180, C181, C182, C183, C184, C185, C186, C187, C188, C189, C19, C20, C21, C210, C211, C212, C218, C22, C220, C221, C222, C223, C224, C227, C229, C23, C24, C240, C241, C248, C249, C25, C250, C251, C252, C253, C254, C257, C258, C259, C26, C260, C261, C268, C269, C30, C300, C301, C31, C310, C311, C312, C313, C318, C319, C32', C320, C321, C322, C323, C328, C329, C33, C340, C341, C342, C343, C348, C349, C37, C380, C381, C382, C383, C384, C388, C390, C398, C399, C400, C401, C402, C403, C408, C409, C410, C411, C412, C413, C414, C418, C419, C430, C431, C432, C433, C434, C435, C436, C437, C438, C439, C440, C441, C442, C443, C444, C445, C446, C447, C448, C449, C450, C451, C452, C457, C459, C460, C461, C462, C463, C467, C4670, C4671, C4672, C4678, C468, C469, C470, C471, C472, C473, C474, C475, C476, C478, C479, C480, C481, C482, C488, C490, C491, C492, C493, C494, C495, C496, C498, C499, C500, C501, C502, C503, C504, C505, C506, C508, C509, C510, C511, C512, C518, C519, C52, C530, C531, C538, C539, C540, C541, C542, C543, C548, C549, C55, C56, C570, C571, C572, C573, C574, C577, C578, C579, C58, C600, C601, C602, C608, C609, C61, C620, C621, C629, C630, C631, C632, C637, C638, C639, C64, C65, C66, C670, C671, C672, C673, C674, C675, C676, C677, C678, C679, C680, C681, C688, C689, C690, C691, C692, C693, C694, C695, C696, C698, C699, C700, C701, C709, C710, C711, C712, C713, C714, C715, C716, C717, C718, C719, C720, C721, C722, C723, C724, C725, C728, C729, C73, C740, C741, C749, C750, C751, C752, C753, C754, C755, C758, C759, C760, C761, C762, C763, C764, C765, C767, C768, C810, C811, C812, C813, C817, C819, C820, C821, C822, C827, C829, C830, C831, C832, C833, C834, C835, C836, C837, C838, C839, C840, C841, C842, C843, C844, C845, C850, C851, C857, C859, C880, C881, C882, C883, C887, C889, C900, C901, C902, C910, C911, C912, C913, C914, C915, C917, C919, C920, C921, C922, C923, C924, C925, C927, C929, C930, C931, C932, C937, C939, C940, C941, C942, C943, C944, C945, C947, C950, C951, C952, C957, C959, C960, C961, C962, C963, C967, C969, C97 |
| Moderate or severe liver disease | B179, I85, I850, I859, I864, I982, I983, K700, K703, K711, K717, K721, K729, K74x, K76x, K704 |
| Metastatic solid tumor | C77x, C78x, C79x, C80x |
| AIDS/HIV | B20x, B21x, B22x, B23x, B24x |

Additional file **1: Table S2: Comparison of patients not admitted in ICU/IntermCU versus the others**

| Variables (Median(IQR)/ N(%)) | ICU/ IntermCU  admission | No ICU/IntermCU admission | | p value | |  |
| --- | --- | --- | --- | --- | --- | --- |
|  | 4128 | 373 | |  | |  |
| Age, median (IQR) | 61 [50 ; 71] | 75 [59 ; 86] | | <0.01 | |  |
| Age > 65 years old | 1667 (40.4) | 249 (66.8) | | <0.01 | |  |
| Sex (Male) | 2307 (55.9) | 169 (45.3) | | <0.01 | |  |
| Diabetes | 758 (18.4) | 66 (17.7) | | 0.75 | |  |
| Cardiovascular disease | 563 (13.6) | 52 (13.9) | | 0.87 | |  |
| Cancer | 504 (12.2) | 52 (13.9) | | 0.33 | |  |
| Chronic liver disease | 420 (10.2) | 23 (6.2) | | 0.01 | |  |
| Chronic kidney disease | 208 (5) | 21 (5.6) | | 0.62 | |  |
| Charlson score, median (IQR) | 1 [0 ; 3] | 1 [0 ; 2] | | 0.37 | |  |
| Number of hospital stays, median (IQR) | 2 [1 ; 2] | 1 [1 ; 2] | | <0.01 | |  |
| Category of first hospital |  |  | |  | |  |
| Regional/universitary hospital | 1791 (43.4) | 105 (28.2) | | <0.01 | |  |
| Local | 1947 (47.2) | 229 (61.4) | | . | |  |
| Other | 390 (9.4) | 39 (10.5) | | . | |  |
| Category of second hospital (miss=2243) |  |  | |  | |  |
| Regional/universitary hospital | 834 (39.5) | 50 (34.5) | | 0.49 | |  |
| Local | 981 (46.4) | 73 (50.3) | | . | |  |
| Other | 298 (14.1) | 22 (15.2) | | . | |  |
| Up category hospital (to regional hospital) (miss=2243) | 234 (11.1) | 14 (9.7) | | 0.6 | |  |
| Volume of first hospital (from 2011 to 2020) |  |  | |  | |  |
| < 5 admissions | 429 (10.4) | 68 (18.2) | | <0.01 | |  |
| Between 5 and 10 | 488 (11.8) | 51 (13.7) | | . | |  |
| Between 10 and 20 | 801 (19.4) | 88 (23.6) | | . | |  |
| Between 20 and 50 | 1170 (28.3) | 92 (24.7) | | . | |  |
| >50 | 1240 (30) | 74 (19.8) | | . | |  |
| Volume of the second hospital (from 2011 to 2020) (miss=2243) | | |  | |  | |
| < 5 admissions | 336 (15.9) | 30 (20.7) | | 0.14 | |  |
| Between 5 and 10 | 299 (14.2) | 25 (17.2) | | . | |  |
| Between 10 and 20 | 358 (16.9) | 29 (20) | | . | |  |
| Between 20 and 50 | 520 (24.6) | 27 (18.6) | | . | |  |
| >50 | 600 (28.4) | 34 (23.4) | | . | |  |
| Increase in Volume category from hospital 1 to 2 (miss=2243) | 491 (11.9) | 27 (7.2) | | <0.01 | |  |
| Transfer to another hospital |  |  | |  | |  |
| Admission from another hospital | 490 (11.9) | 19 (5.1) | | <0.01 | |  |
| Admission from home | 3628 (87.9) | 354 (94.9) | | <0.01 | |  |
| Number of wards, median (IQR) | 3 [2 ; 5] | 2 [2 ; 3] | | <0.01 | |  |
| Number of organ failures (OF) on admission, median (IQR) | 1 [0 ; 3] | 1 [0 ; 1] | | <0.01 | |  |
| Cardiovascular failure on admission | 1437 (34.8) | 70 (18.8) | | <0.01 | |  |
| Hematological failure on admission | 199 (4.8) | 10 (2.7) | | 0.06 | |  |
| Neurological failure on admission | 2109 (51.1) | 125 (33.5) | | <0.01 | |  |
| Renal failure on admission | 565 (13.7) | 35 (9.4) | | 0.02 | |  |
| Respiratory failure on admission | 1635 (39.6) | 11 (2.9) | | <0.01 | |  |
| Septic shock on admission | 960 (23.3) | 5 (1.3) | | <0.01 | |  |
| Number of organ failures during ICU stay | 3 [2 ; 3] | 1 [1 ; 2] | | <0.01 | |  |
| Cardiovascular failure during ICU stay | 2770 (67.1) | 143 (38.3) | | <0.01 | |  |
| Neurological failure during ICU stay | 3631 (88) | 240 (64.3) | | <0.01 | |  |
| Renal failure during ICU stay | 1184 (28.7) | 72 (19.3) | | <0.01 | |  |
| Respiratory failure during ICU stay | 3041 (73.7) | 34 (9.1) | | <0.01 | |  |
| Septic shock during ICU stay | 1986 (48.1) | 16 (4.3) | | <0.01 | |  |
| Purpura | 162 (3.9) | 2 (0.5) | | <0.01 | |  |
| Brain stem failure | 675 (16.4) | 6 (1.6) | | <0.01 | |  |
| Seizure | 503 (12.2) | 35 (9.4) | | 0.11 | |  |
| Coma | 2195 (53.2) | 62 (16.6) | | <0.01 | |  |
| Focal sign without coma | 341 (8.3) | 36 (9.7) | |  | |  |
| No Coma no Focal sign | 1592 (38.6) | 275 (73.7) | |  | |  |
| Vascular Complication | 272 (6.6) | 10 (2.7) | | <0.01 | |  |
| Hydrocephaly | 55 (1.3) | 2 (0.5) | | 0.19 | |  |
| Cardiovascular infection | 324 (7.8) | 7 (1.9) | | <0.01 | |  |
| Respiratory infection | 1444 (35) | 102 (27.3) | | <0.01 | |  |
| Hospital length of stay, median (IQR) | 21 [13 ; 39] | 15 [9 ; 25] | | <0.01 | |  |
| ICU length of stay, median (IQR) | 6 [2 ; 14] | 0 [0 ; 0] | | <0.01 | |  |
| Costs in €, median (IQR) | 39848 [23089 ; 75440] | 15380 [8326 ; 20151] | | <0.01 | |  |
| Discharge home | 1855 (44.9) | 192 (51.5) | | 0.02 | |  |
| Discharge hospital | 1402 (34) | 90 (24.1) | | <0.01 | |  |
| Discharge readaptation | 580 (14.1) | 50 (13.4) | | 0.73 | |  |
| Death | 865 (21) | 89 (23.9) | | 0.19 | |  |

Additional file 1: **Table S3: Univariate analysis of factors associated with death among patients admitted in ICU for pneumococcal meningitis**

| Variables | OR | 95% CI | P-value |
| --- | --- | --- | --- |
| Direct ICU admission | 0.99 | [0.86 ; 1.13] | 0.83 |
| Age > 65 yo | 2.00 | [1.74 ; 2.31] | <0.01 |
| Sex (Female) | 0.85 | [0.73 ; 0.98] | 0.02 |
| Cardiovascular disease | 1.31 | [1.07 ; 1.59] | <0.01 |
| Chronic respiratory disease | 0.87 | [0.66 ; 1.15] | 0.34 |
| Chronic kidney disease | 1.44 | [1.07 ; 1.95] | 0.02 |
| Diabetes | 1.05 | [0.88 ; 1.26] | 0.57 |
| Cancer | 1.44 | [1.18 ; 1.76] | <0.01 |
| Local hospital | 1.28 | [1.09 ; 1.49] | <0.01 |
| Other (private hospital, short-stay hospital) | 1.51 | [1.19 ; 1.91] | 0.08 |
| Regional/universitary hospital | 1 |  | <0.01 |
| Transfer from home | 2.29 | [1.42 ; 3.7] | <0.01 |
| Organ failure on admission |  |  |  |
| Neurological failure | 0.91 | [0.79 ; 1.05] | 0.19 |
| Respiratory failure | 1.41 | [1.22 ; 1.62] | <0.01 |
| Cardiovascular failure | 1.77 | [1.53 ; 2.04] | <0.01 |
| Renal failure | 2.65 | [2.19 ; 3.2] | <0.01 |
| Hematological failure | 2.38 | [1.78 ; 3.18] | <0.01 |
| Endocarditis | 0.94 | [0.72 ; 1.23] | 0.67 |
| Pneumonia | 1.48 | [1.28 ; 1.71] | <0.01 |

ICU : intensive and intermediate care units, Volume admissions of hospital corresponding to the volume of admissions for meningitis

The ICD-10 codes used for definitions are displayed in the Supplementary table S1.

Additional file **1: Table S4: Risk factors for hospital death among patients without organ failure on hospital admission**

| Variables | OR | 95% CI | P-value |
| --- | --- | --- | --- |
| Age > 65 yo | 1.60 | [1.24 ; 2.07] | <0.01 |
| Sex (Female) | 0.74 | [0.57 ; 0.96] | 0.02 |
| Cancer | 1.52 | [1.12 ; 2.07] | 0.01 |
| Local hospital | 1.25 | [0.93 ; 1.66] | 0.13 |
| Other (private hospital, short-stay hospital) | 1.36 | [0.96 ; 1.94] | 0.08 |
| Regional/universitary hospital | 1 |  |  |
| Transfer from home | 2.36 | [0.52 ; 10.62] | 0.26 |
| Pneumonia | 1.28 | [0.98 ; 1.66] | 0.07 |
| Direct ICU admission | 0.76 | [0.48 ; 1.19] | 0.22 |
